# Supplementary material for: Droplet bioprinting of acellular and cell-laden structures at high-resolutions
Source: Biofabrication. Author manuscript; Available in PMC 2025 May 28. (PMC12117511; doi:10.1088/1758-5090/ad4c09)
Supplement: SI_Text [file NIHMS2075418-supplement-SI_Text.pdf]

Droplet bioprinting of acellular and cell-laden structures at high-resolutions

*Puskal Kunwar[#@], Ujjwal Aryal[#@], Arun Poudel[#@], Daniel Fougnier[#@], Zachary J Geffert[#@], Rui Xie[#@], Zhen Li[ψ], Pranav Soman[#@]**

[#]Syracuse University, Biomedical, and Chemical Engineering Department, Syracuse, New York, 13210, USA

[@]BioInspired Institute, Syracuse, New York, 13210, USA

[ψ]Clemson University, Department of Mechanical Engineering, Clemson, SC 29634, USA

Corresponding Author*: e-mail: psoman@syr.edu

1. Comparison of different bioprinting techniques

Bioprinting Methods	Resolution (µm)	Speed	Overall Fidelity
Extrusion	100-500	generally slow	limited in producing fine details
Inkjet based	50-300	moderate	excel where the precise placement of cells is required
Drolet printing	10-100	high	suitable for intricate structures requiring fine details

Table S1. *Comparison of Extrusion printing, Inkjet printing, and Droplet printing in terms of resolution, speed, and overall fidelity.* [1–3]

2. EDS analysis of mineralized structure as compared to a control sample

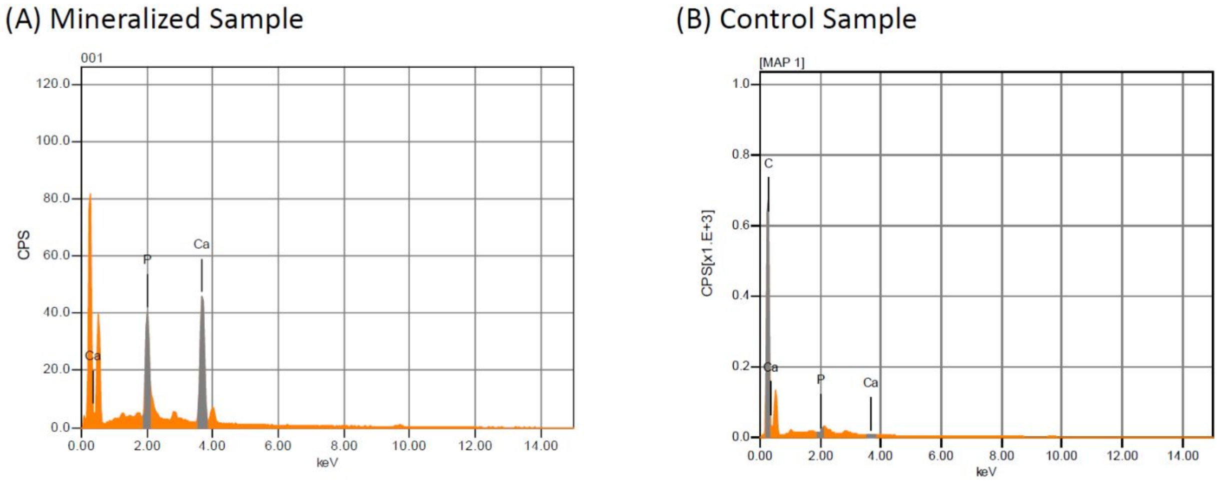

Formula	mass%	Atom%	Sigma
C*	76.38	90.78	0.02
P	7.67	3.53	0.02
Ca	15.95	5.68	0.03
Total	100.00	100.00	

Formula	mass%	Atom%	Sigma
C*	99.92	99.98	0.01
P	nd	nd	
Ca*	0.08	0.02	0.00
Total	100.00	100.00	

Figure S1. (A) *EDS spectrum from the as-fabricated mineralized structure shows the presence of Calcium and Phosphorus peak.* ***(B)*** *EDS spectrum from the control structure (cell free) shows the presence of Calcium and Phosphorus peak.*

Appendix: Many-Body Dissipative Particle Dynamics

The multiphase fluid simulations were performed using an open-source software named Large-scale Atomic/Molecular Massively Parallel Simulator (LAMMPS)[4]. The many-body dissipative particle dynamics model is included in a LAMMPS module DPD-MESO, where a boundary method developed by Li, et al. [5] to apply the no slip boundary condition for arbitrary geometries was used. The simulation results were visualized using a free molecular visualization program named Visual Molecular Dynamics (VMD) [6].

Many-body dissipative particle dynamics (mDPD) method is developed based on the standard DPD framework [7]. The time evolution of mDPD particles i is governed by the conservation of momentum, which is described by the following set of equations,

$$\frac{d\boldsymbol{r}_i}{dt} = \boldsymbol{v}_i \qquad (1)$$

$$\frac{d\boldsymbol{v}_i}{dt} = \boldsymbol{F}_i = \sum_{i \neq j} \left(\boldsymbol{F}_{ij}^C + \boldsymbol{F}_{ij}^D + \boldsymbol{F}_{ij}^R \right) \tag{2}$$

where t, $\boldsymbol{r}_i$, $\boldsymbol{v}_i$and $\boldsymbol{F}_i$ denote time, and position, velocity, force vectors, respectively. The summation of forces is carried out over all other particles within a cutoff radius r_c, beyond which the direct interactions between particles are considered to be zero.

The three components of $\boldsymbol{F}_i$ include the conservative force $\boldsymbol{F}_{ij}^C$, dissipative force $\boldsymbol{F}_{ij}^D$ and random force $\boldsymbol{F}_{ij}^R$, which are expressed as [8]

$$\boldsymbol{F}_{ij}^C = A\omega_c(r_{ij})\boldsymbol{e}_{ij} + B(\rho_i + \rho_j)\omega_d(r_{ij})\boldsymbol{e}_{ij} \tag{3}$$

$$\boldsymbol{F}_{ij}^D = -\gamma\omega_D(r_{ij})(\boldsymbol{e}_{ij} \cdot \boldsymbol{v}_{ij})\boldsymbol{e}_{ij} \tag{4}$$

$$\boldsymbol{F}_{ij}^R = \delta\omega_R(r_{ij})\xi_{ij}\Delta t^{-\frac{1}{2}}\boldsymbol{e}_{ij} \tag{5}$$

where r_{ij} is distance between particles i and j, $\boldsymbol{e}_{ij}$ is the unit vector from particle j to i, and $\boldsymbol{v}_{ij} = \boldsymbol{v}_i - \boldsymbol{v}_j$ is the velocity difference. ω_c, ω_d, ω_D and ω_R are the weight functions of $\boldsymbol{F}_{ij}^C$, $\boldsymbol{F}_{ij}^D$and $\boldsymbol{F}_{ij}^R$, respectively. A negative coefficient $A < 0$ stands for an attractive force and a positive coefficient $B > 0$ results in a density-dependent repulsive force. γ is dissipative parameter, δ is the Gaussian white noise with zero mean and unit variance which describes the degrees of freedom that have been eliminated from the coarse-graining process [9]. The dissipative force and random force act as a thermostat if the dissipation parameter γ and the amplitudes of white noise δ satisfy the fluctuation-dissipation theorem [10] requiring $\delta^2 = 2\gamma k_B T$ and $\omega_D(r) = [\omega_R(r)]^2$, in which k_B is the Boltzmann constant and T is the temperature. A common choice of the weight function is $\omega_c = 1 - r_{ij}/r_c$ and $\omega_D = {\omega_R}^2 = (1 - r_{ij}/r_c)^s$for $r_{ij} \leq r_c$ and vanish for $r_{ij} > r_c$, while $\omega_d = 1 - r_{ij}/r_d$ with a different cutoff radius $r_d < r_c$.

The mDPD system a droplet-based 3D printing model is constructed with parameters $A_{ss} = A_{ll} = -40$, $B = 25$, $\gamma = 18$, $\delta = 6$, $k_B T = 1$, $r_c = 1.0$, $r_d = 0.75$, $s = 0.5$, and A_{sl} changes from -22 to -34 to adjust the wettability of solid surfaces, in which the parameters A_{ss}, A_{ll} and A_{sl} represent the attractive coefficients in Eq. (3) for solid-solid, liquid-liquid and solid-liquid interactions. Varying the strength of attractive interactions between solid and liquid particles can tune the solid-liquid interfacial tension and hence change the wetting contact angles. The mDPD system with an initial meniscus volume of 50 microliters consists of 793,746 particles, in which 169,169 particles form the cross-linked printhead, 198,888 particles being the PDMS substrate, and 425,689 particles representing the liquid meniscus. The parameters in the mDPD model are

calibrated based on the fluid properties of bioink with a viscosity of $4 \, \mathrm{mPa \cdot s}$, and the wetting contact angle $\theta_1$varies from $30°$ to $110°$ for different PDMS base while θ_2 is $29°$ for crosslinked slab.

References

[1] S. Ramesh, O. L. A. Harrysson, P. K. Rao, A. Tamayol, D. R. Cormier, Y. Zhang, I. V. Rivero, *Bioprinting* **2021**, *21*, DOI 10.1016/j.bprint.2020.e00116.

[2] W. L. Ng, X. Huang, V. Shkolnikov, R. Suntornnond, W. Y. Yeong, *Biodes Manuf* **2023**, *6*, 676.

[3] X. Li, B. Liu, B. Pei, J. Chen, D. Zhou, J. Peng, X. Zhang, W. Jia, T. Xu, *Chem Rev* **2020**, *120*, 10793.

[4] A. P. Thompson, H. M. Aktulga, R. Berger, D. S. Bolintineanu, W. M. Brown, P. S. Crozier, P. J. in 't Veld, A. Kohlmeyer, S. G. Moore, T. D. Nguyen, R. Shan, M. J. Stevens, J. Tranchida, C. Trott, S. J. Plimpton, *Comput Phys Commun* **2022**, *271*, DOI 10.1016/j.cpc.2021.108171.

[5] Z. Li, X. Bian, Y. H. Tang, G. E. Karniadakis, *J Comput Phys* **2018**, *355*, 534.

[6] W. Humphrey, A. Dalke, K. Schulten, *VMD: Visual Molecular Dynamics*, **1996**.

[7] R. D. Groot, P. B. Warren, *Journal of Chemical Physics* **1997**, *107*, 4423.

[8] P. B. Warren, *Vapour-Liquid Coexistence in Many-Body Dissipative Particle Dynamics*, **2003**.

[9] Z. Li, X. Bian, B. Caswell, G. E. Karniadakis, *Soft Matter* **2014**, *10*, 8659.

[10] P. Espanol, P. Warren, *EPL* **1995**, *30*, 191.
